# Permissive hypotensive resuscitation in patients with traumatic hemorrhagic shock

**DOI:** 10.1186/s13049-019-0595-5

**Published:** 2019-02-12

**Authors:** Kai-Hung Ho, Yih-Wen Tarng, Yi-Pin Chou, Hsing-Lin Lin

**Affiliations:** 1 Division of Trauma Surgery, Department of Emergency, Kaohsiung Veterans General Hospital, 386, Da-Chung 1st Road, Kaohsiung, 813 Taiwan; 2 Division of Acute Care Surgery, Department of Critical Care Medicine, Kaohsiung Veterans General Hospital, 386, Da-Chung 1st Road, Kaohsiung City, 813 Taiwan; 3 Division of Thoracic Surgery, Department of Surgery, Kaohsiung Veterans General Hospital, 386, Da-Chung 1st Road, Kaohsiung, Taiwan; 4 Department of Orthopaedic, Kaohsiung Veterans General Hospital, 386, Da-Chung 1st Road, Kaohsiung, Taiwan; 5 Department of Cosmetic Science, College of Human Ecology, Chang Gung University of Science and Technology, Kweishan, Taoyuan, Taiwan

## Abstract

We read the article “Risks and benefits of hypotensive resuscitation in patients with traumatic hemorrhagic shock: a meta-analysis” by Natthida Owattanapanich et al. with great interest and found it to be insightful. In our commentary, we propose possible reasons why mortality was lower in the permissive hypotension group and how the need for blood transfusion decreased in this group. No significant difference in acute kidney injury (AKI) was evident among patient groups, possibly because all these patients might have AKI. However, we do agree that permissive hypotension is of considerable benefit to trauma patients and worth further study.

To the Editor:

Sir,

We read the article “Risks and benefits of hypotensive resuscitation in patients with traumatic hemorrhagic shock: a meta-analysis” by Natthida Owattanapanich et al. [1] with great interest. The study provided the evidences of using hypotensive resuscitation for reducing mortality, the need for blood transfusions, the incidence of acute respiratory distress syndrome, and multiple organ dysfunction in traumatic hemorrhagic shock patients without inducing significant acute kidney injury (AKI). Permissive hypotension might be used for critical patients who require transfer to trauma center or pre-operation in extreme situations. Short-term hypotension is acceptably tolerated if the bleeding in the trauma patient is not severe, which signifies that the patient may only have small-artery bleeding rather than large-vessel injury. However, if patients condition continues to deteriorate, intravenous fluids should be immediately administered because such patients are at a high risk of mortality. Therefore, compared with patients not subject to aggressive resuscitation efforts, it is not surprising that the mortality rate is higher among these patients.

In addition, the need for blood transfusion can be reduced in patients who receive appropriate procedures to stop bleeding—time is a critical factor in the process. If the bleeding can be stopped in a short time through timely appropriate treatment, the necessary volume of blood transfused can be reduced, obviating the need for permissive hypotension. Time is also critical in cases of AKI when the patient is in shock, but AKI can occur in any patient experiencing shock regardless of whether they receive aggressive resuscitation. Therefore, it is difficult to compare the outcome of AKI in patients all have AKI. Younger patients are known to recover faster from AKI; therefore, subgroup analysis could be performed on the basis of age.

In permissive hypotension, a trauma patient is treated with restricted fluid resuscitation, and permissive hypotension is a major topic in trauma research recently [2]. Another treatment employed during operation is deliberate hypotension, which is applied during orthopedic surgery [3]. These two treatments aim to control systolic blood pressure, maintaining it within 80–100 mmHg, and to decrease bleeding from the arteries of an injured person or an operative site. Permissive hypotension can be applied preoperatively and perioperatively; however, after bleeding is stopped through invasive interventions, it is necessary to maintain normal blood pressure and sufficient urine output. Permissive hypotension can be applied in trauma patients who respond to resuscitation; however, major trauma patients who do not respond to resuscitation, even aggressive fluid resuscitation, may have higher mortality, and permissive hypotension should not be applied in these patients.

Currently, no consensus strategy governs when or how to administer permissive hypotension during resuscitation. Further study should investigate the acceptable duration of low blood pressure and its tolerability.
